# Association Between Extraversion Personality With the Blood Pressure Level in Adolescents

**DOI:** 10.3389/fcvm.2022.711474

**Published:** 2022-03-03

**Authors:** Xiaohua Liang, Guang Hao, Lun Xiao, Shunqing Luo, Guifang Zhang, Xian Tang, Ping Qu, Rina Li

**Affiliations:** ^1^Department of Clinical Epidemiology and Biostatistics, Children's Hospital of Chongqing Medical University, National Clinical Research Center for Child Health and Disorders, Ministry of Education Key Laboratory of Child Development and Disorders, Chongqing Key Laboratory of Pediatrics, Chongqing, China; ^2^Department of Public Health and Preventive Medicine, School of Medicine, Jinan University, Guangzhou, China; ^3^Disease Control and Prevention Center of Jiulongpo District, Chongqing, China; ^4^Medical General Ward of Children's Hospital of Chongqing Medical University, Chongqing, China; ^5^Plastic Surgery Department of Children's Hospital of Chongqing Medical University, Chongqing, China

**Keywords:** extraversion, systolic blood pressure, diastolic blood pressure, mean arterial pressure, urban-rural differences

## Abstract

**Purpose:**

The association between extraversion personality and high blood pressure (BP) has not been well-studied. This study aims to explore the association between extraversion personality and the BP level in a large sample of adolescents.

**Methods:**

As an ongoing study, 5,246 children aged 6–9 years were recruited using a stratified cluster sampling method in 2014. The extraversion personality trait, assessed by the Eysenck personality questionnaire (EPQ, answered by adolescents) in 2019, was used in the main analysis. A total of 3,407 participants were eligible and included in this analysis.

**Results:**

The EPQ extraversion score was negatively associated with a systolic BP, a diastolic BP, and mean arterial pressure (MAP) in a univariate analysis. After adjusting for other covariates, high extraversion score was negatively associated with systolic BP (β = −0.026; 95% CI = −0.047, −0.004; *p* = 0.002). There was an interaction between regions and the extraversion score on systolic BP (*P*_*interaction*_ = 0.037). The stratified analysis showed that, in rural areas, the extraversion score was negatively associated with systolic BP (β = −0.050; 95% CI = −0.081, −0.016; *p* = 0.004). However, we did find an association between the extraversion score and systolic BP in urban areas (β = 0.000; 95% CI = −0.028, 0.028; *p* = 0.996). Similar results were observed for the extraversion assessed by parents.

**Conclusions:**

We observed that extraversion personality was negatively associated with high BP in adolescents, especially for those who were living in rural areas. Our results suggested that a comprehensive intervention should be implemented to promote psychological health in adolescents.

## Introduction

Essential hypertension, as a well-established risk factor of cardiovascular disease, has become a global disease burden and public health concern (1). It has its roots in childhood (2). Own to the different measurement techniques, definitions, and population, the prevalence of hypertension in children and adolescents ranges from 0.3 to 12.6% (3–5) and is still increasing in developing countries. Blood pressure (BP) in childhood and adolescents could predict the risks of hypertension or cardiovascular disease in young adulthood (3, 4); therefore, identifying the risk factors for BP development from childhood into young adulthood is urgent.

Accompanied by lifestyle changes (such as excessive internet and social media use) (5), family structure (6), and academic depression, the stress in children and adolescents has arisen and become a serious social problem. On the other hand, our previous study found that left-behind children in rural areas have a higher prevalence of hypertension (7), which may partially contribute to a difference in psychological factors between left-behind children and children living with their core family (8). Previous studies have identified socioeconomic status (SES) (9), perinatal factors (10), anthropometric variables, nutrition (11), physical activity (12), obesity, and dyslipidemia as the risk factors for hypertension but not all the risk factors of hypertension have not been identified.

In personality psychology, introversion/extraversion is a dimension with the range between the two extremes, and within this range, there are people who are neither extrovert nor introvert (13). Eysenck suggests a concept that extroverts have fairly strong inhibitory processes but poor excitability. With robust nervous systems, extroverts have a high capacity for accepting stimulation. Hence, the brains of extroverts react to stimuli more slowly and weakly, leading to an inclination toward a strong sensory stimulation. In contrast, introverts have strong stimulatory processes. In terms of the cortex, introverts are inherently more motivated, with brains that react faster and more strongly to stimuli (14). It was reported that high extraversion participants showed a relatively lower cardiovascular response to moderate-intensity social stress (15), which plays a vital role in the development of hypertension (16–19). For example, Wright et al. reported a negative strong relationship between mindfulness and perceived stress in African-American college students (19). Similarly, another cross-sectional study also found that work-related stress was positively associated with cardiovascular risk among employees of a logistics company for safety in communications and flight (17). However, a few studies evaluated the impact of personality characteristics on the BP level (13, 20, 21). For example, Hozawa et al. found that the extraversion score was positively correlated with the systolic BP level (20), whereas the other two studies reported that extroversion personality was negatively associated with the BP level (13, 22). Because earlier studies mainly included a limited sample size in adults or special populations, the results were controversial (13, 20, 22, 23). In this study, we hypothesized that extrovertive personality is negatively associated with high BP in adolescents.

Our study aims to examine the association between extrovertive personality and BP in adolescents who are living in urban and rural areas using cross-sectional data with a large sample size in China.

## Methods

### Patient and Public Involvement

Children and their guardians or the public were not involved in the design, conduct, reporting, or dissemination plans of our research.

### Participants

In 2014, a stratified cluster sampling was used to obtain a representative sample of children aged 6–9 years in Chongqing. The first stage of sampling was to randomly select one rural and one urban county, then two communities per county were randomly selected. Participants who met all the following criteria were recruited: (1) participants aged between 6 and 9 years in 2014, (2) resided in the target region for more than 6 months, (3) did not have serious diseases (e.g., nephropathy, cardiovascular disease, or cancer), and (4) obtained consent from both the parent and children for participation. Finally, 5,246 children who were living in the selected communities were informed and included if they satisfied the inclusion criteria. Demographic information and physical examination data were collected at baseline. As an ongoing study, 4,162 participants were followed up in 2019. In addition to the physical examination, the Eysenck personality questionnaire (EPQ) was filled up in 2019. In this study, 3,407 participants with complete data are included in the analyses (Supplementary Figure 1) and a comparison of the difference between the included and excluded participants is presented in Supplementary Table 1. All works in this study were conducted following the ethical guidelines of the 1964 Declaration of Helsinki and its later amendments (24). The Institutional Review Board at the Children's Hospital of Chongqing Medical University gave its approval for the study (File No.: 2019-86). Informed consent was provided by all participants and parents/guardians.

### Measures

Demographic information, SES (parental education level), and the number of children were collected. Parental education level was measured on a four-point scale ( ≤ 9 years, 9–12, 12–15, and >15 years), and we combined bachelor and master's degrees or above as few parents with master's and doctorate degrees. The degree of pubertal development was surveyed by the visit of the pediatrician and children or parents. The validity and reliability of the questionnaire were assessed and were described in detail in a previous publication (7). The questionnaire was completed by the parent or guardians of children after standard training.

Anthropometric measurements were conducted by well-trained pediatric nurses, and the protocol for these measurements was described in a previous publication (7). A mobile medical ultrasonic machine (models-H300D) was used to measure height and weight, and body mass index (BMI) was calculated as weight/height^2^ (kg/m^2^). BP and heart rate were measured on three separate occasions with an OMRON arm-type electronic sphygmomanometer (HEM7051) using an appropriately sized BP cuff placed on the participants' right arm (7). This study used the hypertension diagnostic criteria described by Jie Mi (25), which are suitable for the growth characteristics of children and teenagers in China. Hypertension (26) was defined as the average clinic measured systolic BP and/or diastolic BP ≥ 95th percentile (based on age, sex, and height percentiles). Mean arterial pressure (MAP) (27) was calculated by the formula of [systolic BP + (2 × diastolic BP)]/3.

Extraversion personality was assessed by the Chinese version of EPQ (28) in which 25 items scored on a two-point scale (for positive items NO = 0 and YES = 1) were adopted for extraversion. High scorers on the extraversion scale indicate sociable, exciting, pleasurable, carefree, and aggressive characteristics. Items in extraversion were normalized to the age- and sex-specific norm of China into a score ranging from 0 to 100 and normalized with a mean of 50 and SD of 10. The EPQ score was used in the main analysis. Also, the extroversion personality of participants was assessed and categorized into introvertive, intermediate, and extrovertive by parents.

### Statistical Analyses

Differences of continuous variables in general characteristics of participants among four groups were assessed using an ANOVA. The χ^2^-test was used to test the difference of categorical variables. A generalized linear model (GLM) was used to analyze the association between extraversion and the BP level, and a multivariable logistic regression model was performed to test the association between extraversion and hypertension. Univariate analyses were performed in Model 1. Model 2 was adjusted for age and sex. Model 3 was further controlled for regions (urban/rural), BMI, puberty, the number of children, and father's education. Also, the interaction between regions and extraversion on BP was examined. The data analysis was conducted using SAS 9.4 software (Copyright 2020 SAS Institute, Inc. Cary, NC, USA). A significant difference was defined by an α-level of 0.05.

## Results

The characteristics of these subjects are presented in Table 1. A total of 3,407 samples were included. The mean age was 11.7 ± 0.6 years, 50.7% were men, and 46.6% were living in a rural area. The EPQ extraversion score was 52.0 ± 12.4 for adolescents living in urban areas and was 46.8 ± 12.8 for adolescents living in rural areas. The overall prevalence of hypertension was 3.6%.

**Table 1:** Participants' characteristics.

**Variables**	**Urban**		**Rural**		***P*-Value for sex**	***P*-Value for region**
	**Male**	**Female**	**Male**	**Female**		
No. of participants	937	881	791	798		
Age, years	11.6 ± 0.6	11.6 ± 0.6	11.8 ± 0.7	11.8 ± 0.7	0.015	<0.001
Puberty, *n* (%)	43 (4.6)	377 (42.8)	31 (3.9)	347 (43.5)	<0.001	0.637
Height, cm	151.6 ± 8.4	152 ± 7.3	150.5 ± 8.1	151.3 ± 7.7	0.023	0.001
Weight (kg)	44.5 ± 11.5	43.1 ± 9.6	43.2 ± 10.6	43.1 ± 10.5	0.024	0.071
BMI (kg/m2)	19.2 ± 3.7	18.5 ± 3.1	18.9 ± 3.5	18.7 ± 3.7	<0.001	0.768
SBP, mmHg	105.6 ± 8.9	103.9 ± 8.7	107.6 ± 9.7	107.2 ± 9.5	0.001	<0.001
DBP, mmHg	61.9 ± 6.6	62.8 ± 6.3	63.5 ± 7.1	64.3 ± 6.8	<0.001	<0.001
MAP (mmHg)	76.4 ± 6.6	76.5 ± 6.5	78.2 ± 7.2	78.6 ± 7.1	0.274	<0.001
≥2 children, n(%)	353 (37.7)	374 (42.5)	560 (70.8)	591 (74.1)	0.006	<0.001
**Father's education**						
~9	205 (21.9)	174 (19.7)	327 (41.3)	327 (41.0)	0.276	<0.001
~12	320 (34.2)	322 (36.5)	275 (34.8)	301 (37.7)		
>12	411 (43.9)	385 (43.7)	189 (23.9)	170 (21.3)		
EPQ extraversion score	51.1 ± 12.5	53.0 ± 12.2	46.2 ± 13.3	47.5 ± 12.3	<0.001	<0.001
**Extrovertive personality by parents**						
Introversive	482 (51.5)	463 (52.6)	423 (53.5)	436 (54.6)	0.283	<0.001
Intermediate	338 (36.1)	303 (34.4)	214 (27.1)	247 (30.9)		
Extrovertive	116 (12.4)	115 (13.1)	154 (19.5)	115 (14.4)		

*BMI, body mass index; SBP, systolic blood pressure; DBP, diastolic blood pressure; MAP, mean arterial pressure; EPQ, Eysenck personality questionnaire*.

**Figure 1:**
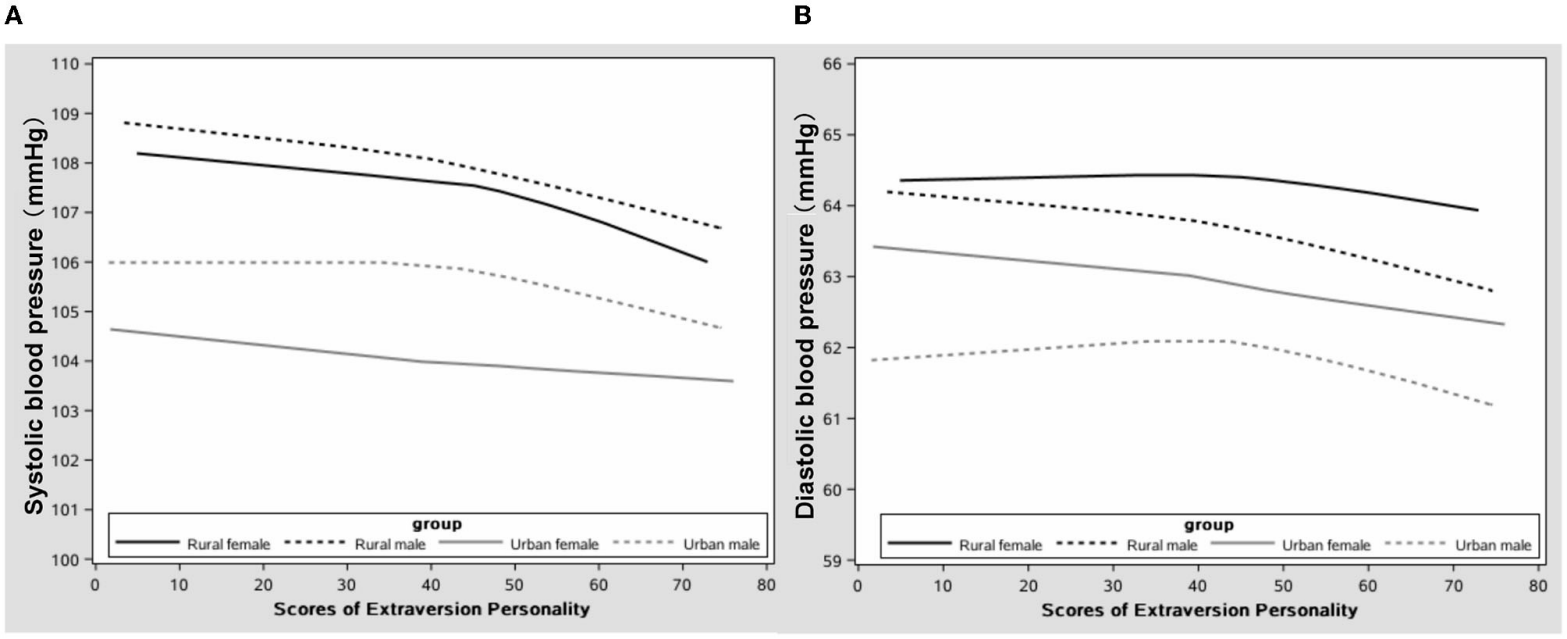
The correlation between extraversion personality and blood pressure in adolescents **(A)** systolic and **(B)** diastolic blood pressure (BP).

**Figure 2:**
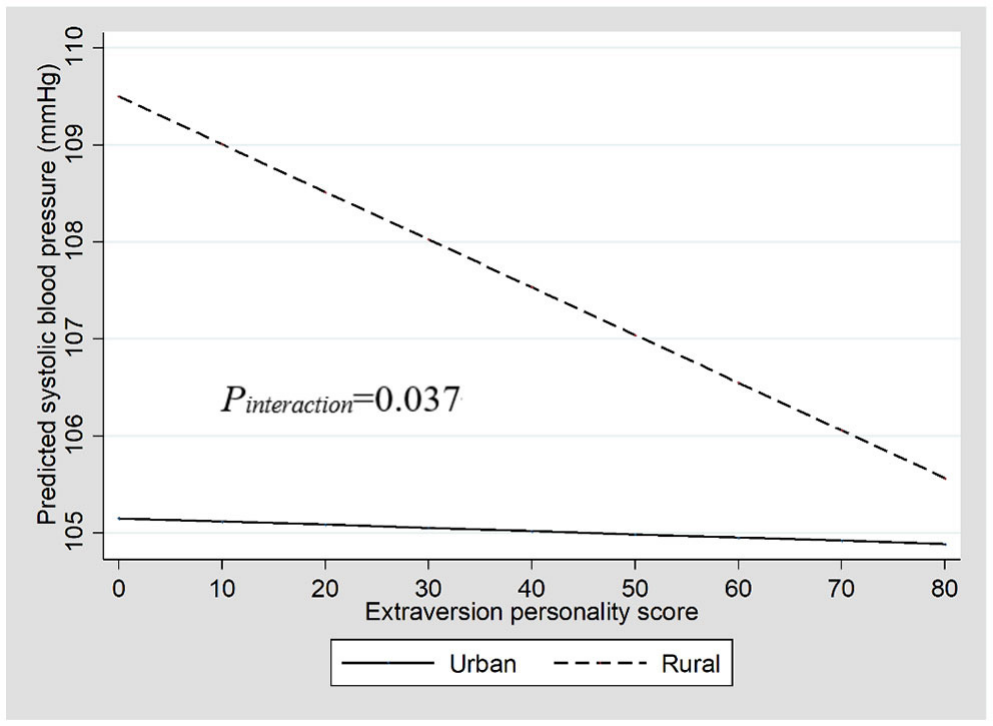
The interaction between extraversion personality and region on a systolic BP.

Relationships of the EPQ extraversion score with a systolic and diastolic BP by region and sex are shown in Figures 1, 2. Systolic and diastolic BP levels were decreased with an increase in the extraversion score. Also, systolic and diastolic BP levels were higher in adolescents living in rural areas than those living in urban areas.

Table 2 shows the relationship of extraversion with BP indexes. The EPQ extraversion score was negatively associated with a systolic BP, a diastolic BP, and MAP in a univariate analysis. After adjusting for age, sex, region, BMI, puberty, the number of children, and father's education, a high extraversion score was negatively associated with a systolic BP (β = −0.026; 95% CI = −0.047, −0.004; *p* = 0.002). Similarly, it was shown that the extraversion personality assessed by parents was negatively associated with a systolic BP (β = −0.457; 95% CI = −0.869, −0.046; *p* = 0.029) and MAP (β = −0.337; 95% CI = −0.658, −0.016; *p* = 0.039). Furthermore, we examined the association between extraversion and hypertension and found similar results (Supplementary Table 2).

**Table 2:** Association between extraversion personality and the blood pressure (BP) level in adolescents.

**Model**	**Self-reported**			**By parents**		
	**β**	**95% CI**	***P-*Value**	**β**	**95% CI**	***P-*Value**
**Model 1**						
SBP	−0.067	−0.091, −0.043	<0.001	−0.509	−0.980, −0.040	0.034
DBP	−0.031	−0.049, −0.014	<0.001	−0.337	−0.679, 0.004	0.053
MAP	−0.043	−0.061, −0.025	<0.001	−0.395	−0.744, −0.046	0.027
Model 2^*^						
SBP	−0.052	−0.076, −0.028	<0.001	−0.414	−0.874, 0.046	0.078
DBP	−0.029	−0.046, −0.011	0.001	−0.320	−0.660, 0.019	0.064
MAP	−0.036	−0.054, −0.019	<0.001	−0.352	−0.696, −0.007	0.046
**Model 3** ^**#**^						
SBP	−0.026	−0.047, −0.004	0.002	−0.457	−0.869, −0.046	0.029
DBP	−0.012	−0.029, 0.006	0.191	−0.277	−0.605, 0.051	0.099
MAP	−0.016	−0.033, 0.001	0.061	−0.337	−0.658, −0.016	0.039

* *Adjusted for age and sex*.

# *Adjusted for age, sex, region, BMI, puberty, number of children, and father's education*.

*SBP, systolic blood pressure; DBP, diastolic blood pressure; MAP, mean arterial pressure*.

There was an interaction between regions and the extraversion score on a systolic BP (*P*_*interaction*_ = 0.037; Figure 2). The stratified analysis showed that, in rural areas, the EPQ score for extraversion was negatively associated with a systolic BP (β = −0.050; 95% CI = −0.081, −0.016; *p* = 0.004). However, we did find an association between the EPQ score for extraversion and a systolic BP in urban areas (β = 0.000; 95% CI = −0.028, 0.028; *p* = 0.996). Similar results were observed for the extraversion assessed by parents (Table 3).

**Table 3:** Association between extraversion personality traits and the BP level by region in adolescents.

**Model**	**Self-reported**			**By parents**		
	**β**	**95% CI**	***P-*Value**	**β**	**95% CI**	***P-*Value**
**Urban**					
SBP	0.000	−0.028, 0.028	0.996	−0.198	−0.728, 0.331	0.463
DBP	−0.009	−0.032, 0.014	0.191	−0.348	−0.783, 0.086	0.116
MAP	−0.006	−0.028, 0.016	0.595	−0.299	−0.715, 0.118	0.160
**Rural**					
SBP	−0.050	−0.081, −0.016	0.004	−0.775	−1.415, −0.135	0.018
DBP	−0.011	−0.038, 0.014	0.388	−0.197	−0.695, 0.301	0.438
MAP	−0.024	−0.050, 0.002	0.067	−0.390	−0.884, 0.104	0.122

*Adjusted for age, sex, region, BMI, puberty, number of children, and father's education*.

*SBP, systolic blood pressure; DBP, diastolic blood pressure; MAP, mean arterial pressure*.

## Discussion

To the best of our knowledge, this is the largest study that explored the relationship between extraversion personality and BP in adolescents. The results revealed that even adjusting for BMI and other covariates, a lower extraversion score was significantly associated with a higher BP in adolescents. Also, we for the first time reported that there was an interaction between regions and the extraversion score on a systolic BP, suggesting an urban-rural difference in the association of extraversion personality with high BP.

Earlier studies on the association between personality traits and hypertension mainly included a limited sample size in adults or a special population. Arkwright et al. found that the prevalence of hypertension was three times more in introvertive male drinkers than in extrovertive male drinkers, but the effect disappeared in drinkers who also smoked (22). Namdar et al. also reported that introvertive healthy participants and hypertensive patients had a higher heart rate, a systolic and diastolic BP than the extrovertive ones, and after music intervention, the extrovertive ones obtained a greater reduction in SBP (13). Consistent with most previous studies, we found that a high extraversion score was positively associated with a lower BP in adolescents.

Besides, our study for the first time revealed that the associations between extraversion personality and hypertension in adolescents were different in the urban and rural areas. As shown in our study, urban adolescents have a higher E score and a higher percentage of extroverts than their rural counterparts. One possible explanation, therefore, is that more left-behind children in rural areas have a lower extraversion score (29) and more psychological imbalance (30, 31), and psychological depression and anxiety (32, 33). Our results suggested that a comprehensive intervention should be implemented to promote psychological health in adolescents, especially for those who were living in rural areas.

The association between the extraversion score and high BP may be explained by different psychosocial stress and anxiety status between introvertive and extrovertive adolescents (34) as an introvert may increase stress (35) and develop greater anxiety (22). Earlier studies found that psychosocial stress and anxiety were associated with an increased risk of hypertension, in which the sympathetic nervous system and renin-angiotensin-aldosterone system may play crucial roles (26–28). Hozawa et al. found that introversion was associated with large differences between screening BP and home BP levels (21). This result was in line with the findings that introversion may represent a hyper-reactive personality, which leads to increased adrenaline concentrations (36) and then to essential hypertension (37). These studies, taken together, suggest that clinical treatment and the prevention of hypertension should consider personality traits.

One major strength of this study is that the association between personality traits and the BP level was examined in a large sample of adolescents. However, our study has one limitation that should be considered when interpreting the results. First, as a cross-sectional analysis, it is difficult to conclude a causal relationship between personality traits and hypertension. However, personality traits tend to be stable over time and consistent across situations (38). Another limitation was that not all risk factors for hypertension were available in this study (39, 40). Finally, our results may not be extrapolated to other countries due to varied national conditions.

In conclusion, this study found that extraversion personality was independently associated with BP, after adjusting for BMI and other covariates. To our knowledge, this is the first study to explore a relationship between extraversion personality traits and BP in a large sample of adolescents. Our study indicated that establishing comprehensive prevention measures (e.g., integrated hospital, community, school, and family) for the development of good personality traits and for providing support for left-behind children in rural areas may help to reduce or prevent hypertension, especially in children who were living in rural areas.

## Data Availability Statement

The original contributions presented in the study are included in the article/Supplementary Material, further inquiries can be directed to the corresponding author/s.

## Ethics Statement

The studies involving human participants were reviewed and approved by the Institutional Review Board at the Children's Hospital of Chongqing Medical University. Written informed consent to participate in this study was provided by the participants' legal guardian/next of kin.

## Author Contributions

XL conceived and designed the experiments. GH and LX performed the experiments. SL, GZ, XT, PQ, and RL participated in the physical measurements. XL and GH wrote the paper. All authors critically reviewed and approved the final paper.

## Funding

This work was supported by the Intelligent Medicine Project (No. NHYX202109), Major Health Project of Chongqing Science and Technology Bureau (No. CSTC2021jscx-gksb-N0001), Research and Innovation Team (No. W0088), National Key Research and Development Project (2017YFC0211705), Education commission of Chongqing Municipality (KJQN201900443), Joint Medical Research Project of Chongqing Municipal Health Commission and Chongqing Science and Technology Bureau (2020MSXM062), National Natural Science Foundation of China (81502826), and Chongqing Medical University Funded Projects (CQMUNCP0204).

## Conflict of Interest

The authors declare that the research was conducted in the absence of any commercial or financial relationships that could be construed as a potential conflict of interest.

## Publisher's Note

All claims expressed in this article are solely those of the authors and do not necessarily represent those of their affiliated organizations, or those of the publisher, the editors and the reviewers. Any product that may be evaluated in this article, or claim that may be made by its manufacturer, is not guaranteed or endorsed by the publisher.
