# Supplementary material for: Association Between Extraversion Personality With the Blood Pressure Level in Adolescents
Source: Front Cardiovasc Med. 2022 Mar 3;9:711474. doi: 10.3389/fcvm.2022.711474 (PMC8927655; doi:10.3389/fcvm.2022.711474)
Supplement: Supplementary file 1 [file Table_1.DOCX]

| STable 1. Comparation between included and excluded participants | | | |
| --- | --- | --- | --- |
|  | Included | Excluded | *P* value |
| Age, years | 11.7(11.7-11.7) | 11.7(11.7-11.8) | 0.119 |
| Sex, females | 49.3(47.6-51.0) | 41.9(38.4-45.4) | <0.001 |
| BMI (kg/m^2^) | 18.8(18.7-18.9) | 19.0(18.8-19.3) | 0.209 |
| Presented by mean or frequency with 95%CI | | | |
