# Supplementary material for: Association Between Extraversion Personality With the Blood Pressure Level in Adolescents
Source: Front Cardiovasc Med. 2022 Mar 3;9:711474. doi: 10.3389/fcvm.2022.711474 (PMC8927655; doi:10.3389/fcvm.2022.711474)
Supplement: Supplementary file 2 [file Table_2.DOCX]

| STable 2 Association of extraversion personality with hypertension in adolescents | | | | |
| --- | --- | --- | --- | --- |
|  |  | OR | 95%CI | *P* value |
| Model 1 |  |  |  |  |
| Extraversion using EPQ |  | 0.980 | 0.969, 0.991 | 0.001 |
| Extraversion from parents |  | 0.706 | 0.561, 0.888 | 0.003 |
| Model 2^*^ |  |  |  |  |
| Extraversion using EPQ |  | 0.988 | 0.976, 1.000 | 0.064 |
| Extraversion from parents |  | 0.706 | 0.560, 0.889 | 0.003 |
| Model 3^#^ |  |  |  |  |
| Extraversion using EPQ |  | 0.988 | 0.976, 1.000 | 0.064 |
| Extraversion from parents |  | 0.724 | 0.568, 0.922 | 0.009 |
| Adjusted for age, sex, region, BMI, puberty, numbers of children, father’s education  OR=odds ratio; CI= confidence intervals | | | | |
