# Supplementary figures and images for: Association Between Extraversion Personality With the Blood Pressure Level in Adolescents
Source: Front Cardiovasc Med. 2022 Mar 3;9:711474. doi: 10.3389/fcvm.2022.711474 (PMC8927655; doi:10.3389/fcvm.2022.711474)

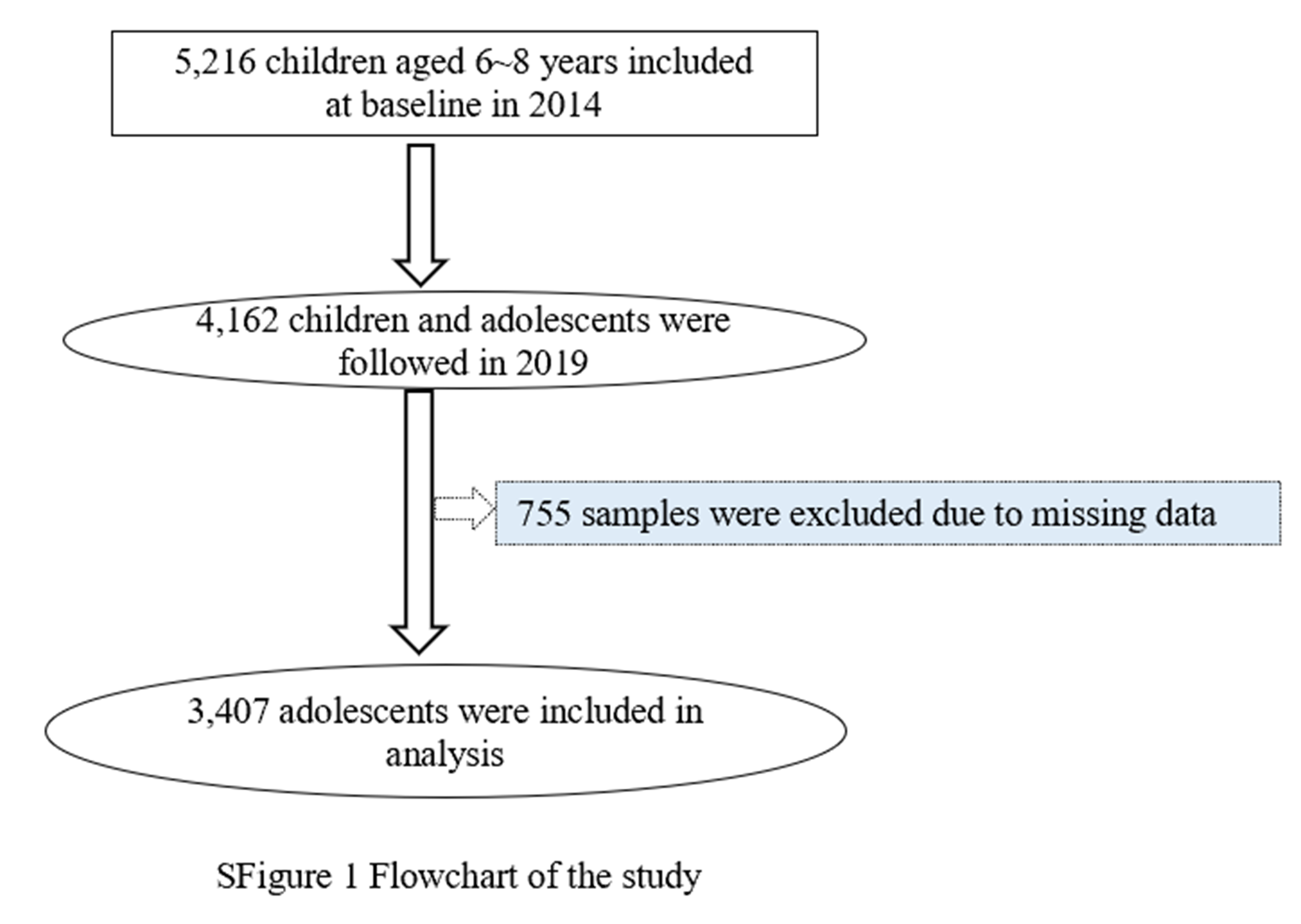

Supplement: Supplementary file 3 [file Image_1.TIF]
